# 9th Annual Conference on New and Re-emerging Infectious Diseases

**DOI:** 10.3201/eid1301.061158

**Published:** 2007-01

**Authors:** Brenda A. Wilson, Uriel Kitron

**Affiliations:** * University of Illinois at Urbana-Champaign, Urbana, Illinois, USA

**Keywords:** infectious diseases, zoonoses, emerging diseases, Bordetella pertussis, Helicobacter pylori, monkeypox, Trypanosoma cruzi, Chagas disease, conference summary

9th Annual Conference on New and Re-emerging Infectious Diseases Urbana, Illinois, USA April 13–14, 2006

The 9th Annual Conference on New and Re-emerging Diseases featured 8 speakers, a poster session, and a panel discussion. The conference was sponsored by the following units of the University of Illinois at Urbana-Champaign: Center for Zoonoses Research; Department of Pathobiology; Environmental Council; Host-Microbe Systems Theme of the Institute for Genomic Biology; Office of International Programs and Studies; Program in Arms Control, Disarmament, and International Security; and Conservation Medicine Center of Chicago. Proceedings are available at http://www.cvm.uiuc.edu/idc

Jonathan Patz, University of Wisconsin-Madison, opened the conference with a presentation about the effects of deforestation and climate on infectious diseases in the northern region of the Peruvian Amazon. Concomitant with new deforestation, the area experienced a sharp (50-fold) rise in malaria incidence during 1987–1997, coupled with an invasion by Anopheles darlingi, the most important vector of malaria in South America. A strong relationship has been found between the extent of deforestation, larval sites of A. darlingi, and abundance of adult mosquitoes. The need for a broad-based policy approach to ecology-disease relationships was highlighted.

Paul Gibbs, University of Florida-Gainesville, discussed emerging infectious disease trends. The potential overshadowing of recent, naturally occurring emerging diseases and epidemics (i.e., foot-and-mouth disease, avian and canine influenza, monkeypox, severe acute respiratory syndrome, bluetongue, bovine spongiform encephalopathy, and West Nile encephalitis) by a succession of disasters, both natural and manmade, was examined. Dr. Gibbs presented the global factors that have led to the increased emergence of these diseases, a summary of lessons learned, and a broad agenda for action.

Alison Weiss, University of Cincinnati, addressed the reemergence of Bordetella pertussis as a notable health threat despite active vaccination programs. She discussed the increased cases of pertussis during the past 25 years and the shift from a whole-cell vaccine to acellular pertussis vaccines, which failed to reverse this increase. Bactericidal immunity, if achievable, was presented as an option for preventing the disease (whooping cough) and transmission of the agent (B. pertussis).

Steven Blanke, University of Illinois at Urbana-Champaign, discussed persistence mechanisms of Helicobacter pylori and explored the hypothesis that phase variation results in altered forms of H. pylori that can withstand the gastric environment. Phase variants of a single gene have been found to demonstrate remarkably distinct properties in terms of stress resistance and virulence factor expression.

R. Mark Buller, Saint Louis University, presented evidence that human monkeypox is a poxvirus zoonotic disease. The first US outbreak of monkeypox virus infection in 2003, unlike previous outbreaks in Africa, resulted in no deaths and no documented human-to-human transmission. Evidence supports the existence of monkeypox virus strains that differ in virulence and transmissibility for humans.

Ricardo Gurtler, University of Buenos Aires, outlined a longitudinal study of the transmission dynamics and control of Trypanosoma cruzi, the causative agent of Chagas disease, in 3 rural villages in northwestern Argentina. Regular monitoring of insect infestations and parasite infections of triatomine bugs was accompanied by demographic assessments of prevalence and infection in humans and dogs before and after the establishment of community-based surveillance in 1992.

May Berenbaum, University of Illinois at Urbana-Champaign, discussed biological invasions that resulted in establishment of nonindigenous species, their role in contemporary global change, and their subsequent economic and ecologic costs. Effects on health are disproportionately large, in part because invasive species often owe their success to unique chemical defense mechanisms and because, without coevolved enemies of their own, they can serve as extraordinarily effective disease vectors.

Nina Marano, Centers for Disease Control and Prevention, addressed the need for multidisciplinary public policy responses to emerging zoonoses. In particular, she examined the case of highly pathogenic avian influenza in Southeast Asia and presented recommendations for integrated research approaches.

A panel consisting of the conference speakers and local experts explored differences between molecular and ecologic studies, between animal and human studies, and between science and public policy. The panel examined strategies for bridging these gaps. Recommendations from this session will appear in a forthcoming Center for Zoonoses Research white paper.

